# Thyroid Carcinoma Showing Thymic-Like Differentiation Causing Fracture of the Trachea

**DOI:** 10.1155/2016/7962385

**Published:** 2016-03-27

**Authors:** Aikaterini Marini, Meletios Kanakis, Konstantinos Valakis, Nikolaos Laschos, Maria Chorti, Achilleas Lioulias

**Affiliations:** ^1^1st Department of Internal Medicine, Sismanoglio General Hospital of Athens, 15126 Athens, Greece; ^2^Department of Thoracic Surgery, Sismanoglio General Hospital of Athens, 15126 Athens, Greece; ^3^Intensive Care Unit, Sismanoglio General Hospital of Athens, 15126 Athens, Greece; ^4^Department of Pathology, Sismanoglio General Hospital of Athens, 15126 Athens, Greece

## Abstract

Thyroid carcinoma showing thymic-like differentiation (CASTLE) comprises a rare neoplasm of the thyroid gland which arises from ectopic thymic tissue or remnants of brachial pouches. CASTLE is regarded as an indolent neoplasm with a favorable prognosis, irrespective of its metastatic potential. Diagnosis is difficult as clinicopathological features have not been yet well-defined. Radiological findings are not specific and only immunohistochemical positivity for CD5 and CD117 staining is highly suggestive of CASTLE. Despite lack of universally accepted treatment recommendations, the mainstay treatment includes thyroidectomy and systematic lymph node dissection. We report a case of CASTLE tumour with very uncommon characteristics developed in a 76-year-old man, who presented with rapidly deteriorating dyspnea and severe cough, resulting in respiratory failure. At surgery, a suspicious looking tumour arising from the upper pole of the right lobe of the thyroid gland, surrounding the trachea and displacing the right common carotid artery, was identified. The patient underwent en bloc resection of the tumour with the thyroid gland and regional lymph node dissection. This is the first reported case of CASTLE causing tracheal ring fracture.

## 1. Introduction

Tumours of the thymus account for less than 1% of all neoplasms and although they do not contribute significantly to the overall human cancer burden, they are regarded as highly interesting. Their aetiology is largely unknown and their biology is complex. Carcinoma showing thymic-like differentiation (CASTLE) comprises a rare tumour of the thyroid gland arising from ectopic thymic tissue or remnants of brachial pouches [1]. Less than 50 cases have been described in the English literature to date [2]. First described in 1981 by Miyauchi et al., [3] as an intrathyroidal epithelial thymoma, CASTLE was later denominated as carcinoma showing thymic-like elements with specific clinical and pathological features [4]. According to the current World Health Organisation (WHO) classification, CASTLE is an independent clinicopathologic entity of thyroid tumours bearing histologic and immunophenotypic resemblance to thymic carcinoma [5, 6]. We describe a unique case of a patient diagnosed with an aggressive CASTLE, which compressed the trachea causing a tracheal ring fracture.

## 2. Case Report

**Figure 1:**
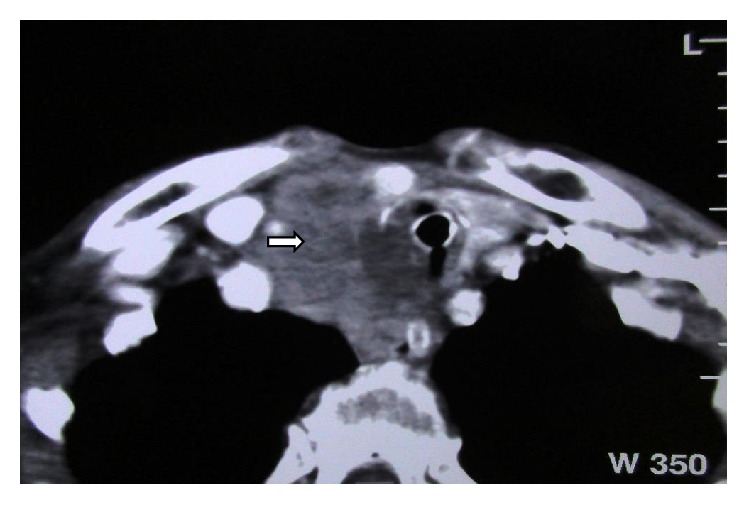
Computed tomography showing the cervical CASTLE tumour surrounding the trachea anterolaterally and invading the thyroid gland.

**Figure 2:**
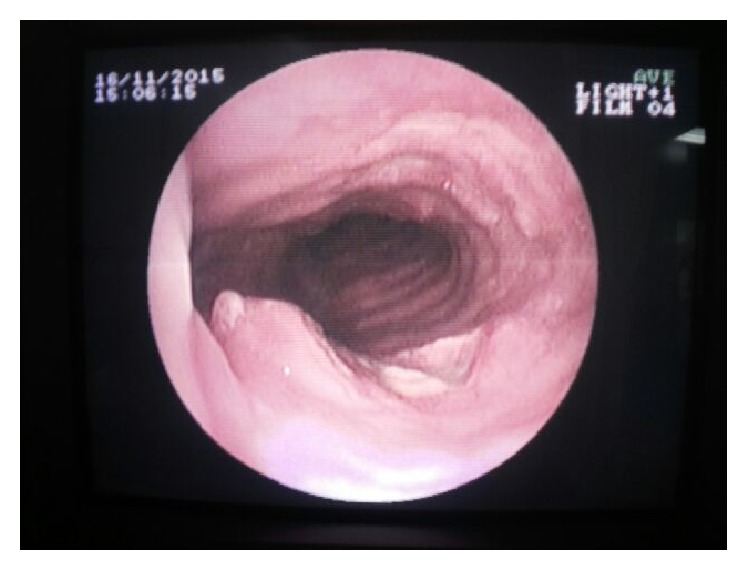
Snapshot from bronchoscopy depicting fracture of the first tracheal ring.

A 76-year-old man was referred to our hospital for investigation of a 20-day history of rapidly deteriorating dyspnea and severe cough. This was associated with dysphagia and cervical pain. The patient was an ex-marble craftsman and an active smoker. His medical history included chronic pulmonary obstructive disease (COPD). A chest X-ray (CXR) showed an imprint at the right side of the trachea. Thyroid ultrasound (US) revealed a large and heterogenous thyroid gland and the presence of a calcified mass, 4.76 × 4.01 cm with high blood perfusion in the right upper lobe of the gland. A contrast-enhanced computed tomography (CT) scan of the neck and thorax reported the corresponding lesion as heterogenous, with irregular borders, extending from the sternoclavicular joints to the upper borders of the thyroid gland. The mass was surrounding the trachea anterolaterally displacing it to the left and the right common artery to the right (Figure 1). There were no enlarged lymph nodes. Bronchoscopy revealed a fracture of the first tracheal ring but there was no evidence of tumour invasion (Figure 2). Cytology from bronchial lavage was negative for cancer cells. Laboratory tests were within normal limits with the exception of elevated D-dimers (2,994 *μ*g/L) and elevated white blood cells (19.27 K/*μ*L). Tumour markers were within normal range values.

On admission, the patient was intubated, due to acute respiratory failure, and he was admitted to the intensive care unit (ICU). Subsequently, he underwent an emergency procedure and a collar incision was carried out. At surgery, a suspicious looking tumour, which was in continuity with the superior pole of the right thyroid lobe, the sheath of right common carotid artery, and the anterolateral portion of the cartilaginous trachea, was identified. Macroscopically, the tumour looked whitish and scleroelastic with moderately regular borders and was firmly adhered to the aforementioned structures. After the right recurrent laryngeal nerve was identified, the tumour was carefully dissected off the right thyroid lobe and the adventitia of the right common carotid artery. A similar approach was carried out to dissect the tumour off the left lobe; hence, an en bloc tumour resection with total thyroidectomy and a regional lymph node dissection was performed. Rapid frozen section biopsy of the tumour in the right thyroid lobe proved to be malignant with features of squamous cell carcinoma and negative surgical margins.

Postoperatively, the patient experienced swallowing difficulty and hoarseness and a tracheostomy was performed on day 2.

**Figure 3:**
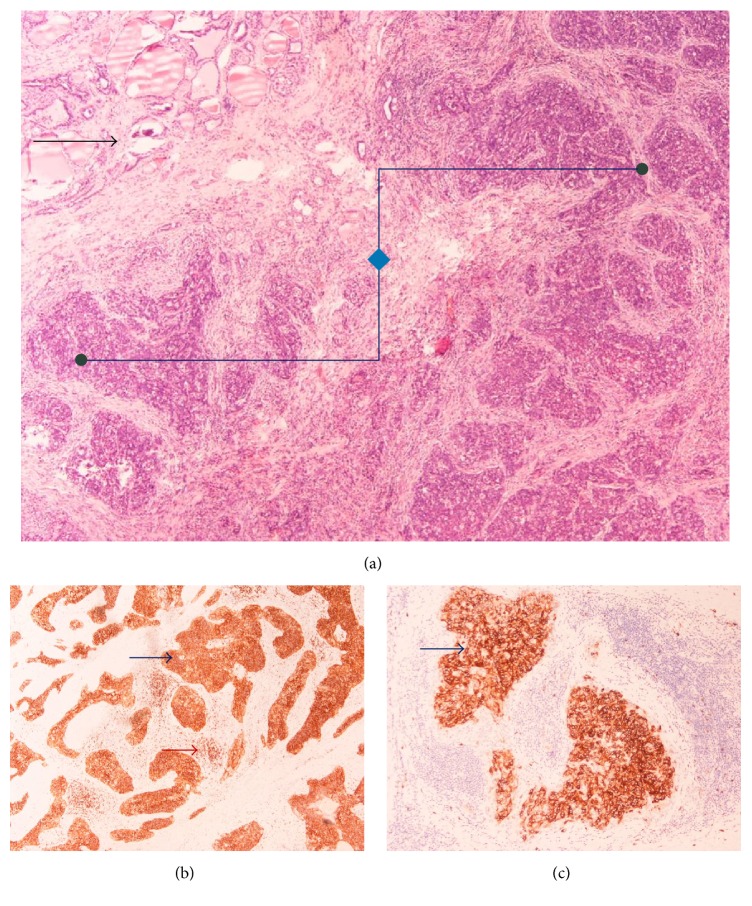
Pathology of the tumour. (a) Thymic carcinoma (right part of picture/angular line) and infiltrated thyroid gland (upper left quadrant of the picture/black arrow) (H&E stain, ×40), (b) Cd5 expression in thymic carcinoma cell (blue arrow) and in lymphocytic T-cell thymic population (red arrow) (IHC, ×100), and (c) CD117 expression in thymic carcinoma (arrow) (IHC, ×100).

**Figure 4:**
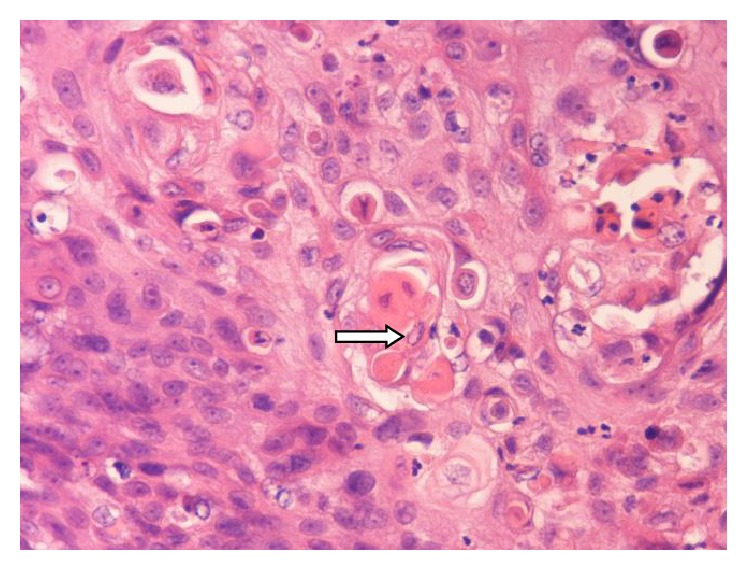
Pathology of the tumour. Thymic squamous cell carcinoma with focal keratinization (arrow): IHC ×400.

**Figure 5:**
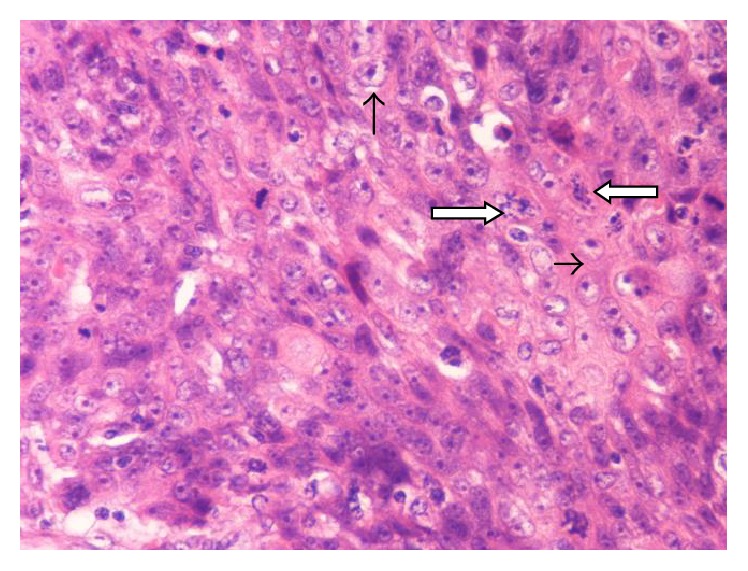
Pathology of the tumour. Thymic squamous cell carcinoma with eosinophilic cytoplasm (horizontal arrow) and vesicular nuclei with nucleoli (vertical arrow). Note atypical mitoses (thick arrows), IHC ×400.

Final pathology examination confirmed frozen section's diagnosis of a squamous cell carcinoma invading the right lobe of the thyroid gland, as well as extracapsular structures of the left lobe. Microscopically, the tumour was described as nonencapsulated, irregularly lobulated, with broad fibrous septa, and lymphoplasmacytic, eosinophilic, histiocytic, and multinuclear infiltration. The neoplastic cells were largely polyhedral or oval with eosinophilic or amphophilic cytoplasm, arranged in islands or nests that showed focal keratinization. The nuclei were vesicular or hyperchromatic with nucleoli. A panel of immunohistochemical stains was performed; the tumour was positive for p63, CD117, CKH, CD5, and CD3 in the lymphocytic part (Figures 3, 4, and 5). It was also negative for the expression of TTF1.

The patient subsequently improved and was discharged home on postoperative day 20. The patient did not receive any additional treatment. 

## 3. Discussion

Tumours with thymic differentiation show partial or complete resemblance to the fetal, mature or involuted thymus or to thymomas. They present as four distinct clinicopathological entities; the ectopic hamartomatous thymomas and ectopic cervical thymomas are regarded as benign as they share common histological features with intrathymic thymomas while the spindle epithelial tumours with thymus-like differentiation (SETTLE) and carcinomas showing thymic-like differentiation (CASTLE) are considered malignant [4, 7].

CASTLE comprises a very rare entity, accounting for 0.1–0.15% of all thyroid cancers [8]. It commonly occurs during the fourth and fifth decades of life and has a slight female predominance (1 : 1.3) [7, 9, 10]. It is thought to originate from ectopic thymus or rudimentary brachial pouches along the thymic line, in or adjacent to the thyroid, which retain the potential to differentiate [7, 11]. Therefore, the lesions arising from ectopic CASTLE may occur in the parapharyngeal space, carotid and posterior spaces [12], and the subcutaneous tissues of head and neck [6]. CASTLE is more frequently found in the left lobe (49%) and the lower poles of the thyroid gland (73–92%) [2, 10, 11]. Only 2% of these tumours tend to localise in its upper parts [2].

The most common subjective symptoms include swallowing difficulties, resulting from palpable neck masses with poor mobility (88%), and hoarseness secondary to recurrent laryngeal nerve paralysis (11%) [2, 11]. Given that the clinical features of CASTLE are nonspecific, invasion to adjacent soft tissue (60%) and metastases to regional lymph nodes (50%) are already present at diagnosis [4, 6, 9, 10]. Brain, liver, and lungs are common metastatic sites too [4]. In some series, the tumour involves the recurrent laryngeal nerve (50%), the adjacent muscles (21%), the oesophagus (10–17%), and the sternum (10%) and only 3–8% of tumours invade the carotid artery [2, 11]. The incidence of tracheal invasion is 24–38%. CASTLE tends to localise mainly in the lower part of the cervical trachea as it appears to arise from the lower poles of the thyroid gland [9]. This distribution results in respiratory symptoms, including bloody sputum and dyspnea [2, 9]. In our case, the trachea was strangled by the tumour anterolaterally, which resulted in a tracheal fracture (Figure 1). To our knowledge, this is the first reported case of a fracture of the tracheal rings due to a CASTLE tumour.

Despite the metastatic potential, CASTLE is regarded as an indolent neoplasm with a favorable prognosis [4, 9, 12–14]. The 5- and 10-year cause-specific survival rates are 90% and 82%, respectively [11]. Macroscopically, CASTLE typically presents with unclear borders, lobulated or expansive growth, fibrous septa, grayish white color, and hard texture [6]. Microscopically, it shows indistinct cell borders and large vesicular nuclei and is composed of variably sized epithelial cell nests separated by dense fibrous septa with many lymphocytes and plasma cell infiltration [6]. The tumour cells show squamoid characteristics with eosinophilic cytoplasm. The pathological differential diagnosis includes thyroid squamous cell carcinoma, papillary or medullary thyroid carcinoma with squamous cell differentiation, and anaplastic thyroid cancer [4, 6]. However, distinguishing CASTLE from these aggressive neoplasms is important as CASTLE has a comparatively favorable prognosis [6].

Nevertheless, final diagnosis is made by immunohistochemistry, which positively stains CASTLE cells for CD5, p63, and cytokeratin and negatively stains CASTLE cells for thyroglobulin, thyroid transcription factor-1 (TTF1), and calcitonin [6, 9, 15]. The expression of marker CD5 by CASTLE cells, along with Hassall's corpuscles, may be highly characteristic of the tumour [6]. The sensitivity and specificity of CASTLE diagnosis by immunohistochemical staining with CD5 are 82% and 100%, respectively [11, 16, 17]. P63, a p53-homolog nuclear transcription factor, is a novel marker for CASTLE disease too [18].

Radiology for CASTLE is not specific. Thyroid ultrasound (US) examination may reveal a lobulated solid hypoechoic mass, with moderate vascularity, which lacks calcifications. The mass usually arises from the lower part of the thyroid and only the slow enlargement of such lesion may raise suspicion for CASTLE [11]. In our case, US showed a large and heterogenous thyroid gland and a calcified mass with high blood perfusion in the right upper lobe of the gland, which was inconclusive for CASTLE. The nonenhanced CT often shows a soft tissue density with unclear border, while contrast-enhanced CT shows slight enhancement by the lesion [6]. In line with the above, our patient's mass was described as heterogenous with irregular borders.

Although the optimal treatment strategy remains uncertain, thyroidectomy and curative surgery, including resection of organs to which the tumour has extended, and systematic lymph node dissection should always be performed [19]. The role of postoperative radiotherapy (RT) is still controversial. Many authors suggest that CASTLE with tumour-negative lymph nodes renders low risk for recurrence and thus surgery without adjuvant radio- or chemotherapy is sufficient [6, 7, 11]. However, it seems that postoperative adjuvant radiotherapy generally prevents tumours of thymic origin from local recurrence, especially those with positive margins [20]. Nevertheless, it is difficult to define the role of adjuvant RT for completely resected invasive tumours presenting with thymic characteristics. Our patient received no radio- or chemotherapy after the thyroidectomy.

## 4. Conclusion

Up to date, less than 50 cases of CASTLE have been reported in English literature [2, 10, 21, 22]. WHO has recently designated CASTLE as an independent type of thyroid carcinoma [8]. Herein, we report on this growing, yet very small, literature, based on a rare case of CASTLE from every aspect. This is the first study of this entity describing a fracture of the trachea due to compression by a CASTLE tumour. The uncommon characteristics of this tumour included the sex and advanced age of the patient as well as the fact that the tumour was arising from the upper pole of the right lobe involving the trachea and the right common carotid artery. CASTLE should be therefore included in the differential diagnosis of patients presenting with a combination of swallowing difficulties and respiratory distress.
